# Editorial: Motor interventions: balance and cognition in older individuals

**DOI:** 10.3389/fnhum.2024.1516396

**Published:** 2024-11-19

**Authors:** Gustavo Christofoletti, Warren G. Darling

**Affiliations:** ^1^Institute of Health, Faculty of Medicine, Federal University of Mato Grosso do Sul, Campo Grande, Brazil; ^2^Department of Health and Human Physiology, University of Iowa, Iowa City, IA, United States

**Keywords:** motor interventions, cognitive interventions, balance, cognition, older individuals

Over the past few decades, the number and profile of individuals aged 65 years or older has changed significantly. Worldwide there are now over 800,000,000 people age 65 years or older and the percentage of older age people in the population has doubled from 5 to 10% since 1960 (United Nations, 2022). In the past, advanced age was associated with functional incapacity. Today, older individuals are physically active and independent, and have busy schedules filled with work and leisure activities. Nowadays, older adults live longer and enjoy a better quality of life than they did in past decades (Marzo et al., 2023). Understanding the mechanisms underlying methods to maintain independence in performing daily activities and good mental and physical health is more important than ever because of our aging population.

Despite living longer on average and with better health, many older individuals do still suffer from aging. Recent studies have indicated that aging is associated with a range of micro- and macrostructural alterations in the brain, leading to reduced cortical thickness, decline in gray matter volume, and changes in white matter that are associated with declines in mental and physical function (e.g., Turrini et al., 2023; Zheng et al., 2019; Demnitz et al., 2017). Acute and chronic neurological conditions, such as stroke and Parkinson's disease, can have devastating effects on quality of life by impairing balance and mobility as well as cognitive function (e.g., Björck et al., 2024; Candel-Parra et al., 2022). However, to combat both healthy and pathological aging, older adults are now more physically active than before and place greater emphasis on physical activity, cognition, and mental and social health to maintain a high quality of life (see Dogra et al., 2022 for a review).

The term “motor intervention” is used by physical therapists, physical education professionals, occupational therapists, speech therapists, and other specialists to refer to a wide range of exercise interventions focused on movement. In the past these interventions primarily aimed to improve strength and motor function in individuals of all ages. Historically these interventions focused on outcomes such as balance, mobility and prevention of falls which can cause devastating injuries such as hip fractures and head injuries especially in older populations. Importantly, recent studies have expanded the scope of exercise intervention research, highlighting its additional benefits for cognition and mental health (e.g., Zhu et al., 2024).

Publications on motor interventions for older adults have increased substantially in recent decades, reflecting the growing recognition of its importance in this population. A quick PubMed search using the keywords “motor intervention” OR “movement” OR “exercise” AND “older adults” AND “1950 – 1960 (Date – Entry)” identified only 45 studies on motor intervention in older adults published between 1950 and 1960. For the period between 2010 and 2020, the search returned 165,849 studies using the same keywords.

In this Research Topic we aimed to explore the benefits of motor interventions on balance and cognition in community-dwelling older adults. We have received important contributions from researchers in the United States (Stegemöller et al.), China (Liu et al.; Zhibo et al.), Germany, Austria, and Switzerland (Lohmann et al.). The collected publications explored the effects of motor interventions in healthy older and diseased populations.

The first study included in this Research Topic was conducted by Stegemöller et al.. They compared the effects of auditory cueing (on one or both sides) on toe tapping and gait responses in individuals with Parkinson's disease. The findings are noteworthy, suggesting that cueing on both sides yields better results than cueing on only one side. This study also identified a significant relationship between toe tapping to auditory cueing and gait performance in people with Parkinson's disease.

The second study assessed effects of motor imagery therapy in individuals with stroke (Liu et al.). The results demonstrated that motor imagery therapy combined with traditional therapy can enhance upper limb motor function and improve independence in performing daily activities in patients with stroke. They also observed increased interaction between sensorimotor and cognitive networks with motor imagery training, which may have additional benefits on cognitive function. This finding is important for specialists seeking improved rehabilitation options for stroke.

Finally, we had two systematic reviews with meta-analyses in our Research Topic. The first study assessed evidence for implementing stretching to enhance balance across individuals of all ages (Lohmann et al.). The findings revealed certain benefits of stretching for balance, but the authors warned about potential biases in some studies, underscoring the low certainty of evidence. The second study explored the benefits of Tai Chi, a traditional Chinese motor and meditative intervention, on balance in community-dwelling healthy older adults (Zhibo et al.). The findings were encouraging as Tai Chi interventions were effective in improving balance and mobility, which should contribute to improved independence and better physical and mental health. Notably, increasing the frequency and duration of Tai Chi sessions resulted in greater improvements in balance and mobility.

Together, these four studies offer an important overview of motor interventions in older adults. We invite readers to assess these studies, as they present diverse approaches that are significant for this population and for health care professionals.
